# Short hairpin-loop-structured oligodeoxynucleotides reduce HSV-1 replication

**DOI:** 10.1186/1743-422X-6-43

**Published:** 2009-04-27

**Authors:** Alexander Falkenhagen, Jochen Heinrich, Karin Moelling

**Affiliations:** 1 University of Zurich, Gloriastrasse 30/32, CH-8006 Zurich, Switzerland; 2 Institute for Advanced Study, Wallotstraße 19, D-14193 Berlin, Germany

## Abstract

The Herpes simplex virus (HSV) is known as an infectious agent and widespread in the human population. The symptoms of HSV infections can range from mild to life threatening, especially in immune-compromised individuals. HSV infections are commonly treated with the guanosine analogue Aciclovir, but reports of resistance are increasing. Efforts are made to establish single-stranded antisense oligodeoxynucleotides (as) and small interfering ribonucleic acids (siRNAs) for antiviral treatment. Recently, another class of short interfering nucleic acids, partially double-stranded hairpin loop-structured 54 mer oligodeoxynucleotides (ODNs), was shown to allow hydrolysis of HIV RNA by binding to the viral RNA. This leads to a substrate for the viral RNase H. To assess the potential of such ODNs for inhibition of HSV-1 replication, five partially double-stranded ODNs were designed based on the sequences of known siRNAs against HSV-1 with antiviral activity. Three of them are directed against early and two against leaky late genes. Primary human lung fibroblasts, MRC-5, and African green monkey kidney cells, Vero, were transfected with ODNs and subsequently infected. The effect on HSV-1 replication was determined by analyzing the virus titer in cell culture supernatants by quantitative PCR and plaque assays. An inhibitory effect was observed with all five selected ODNs, with two cases showing statistical significance in both cell types. The observed effect was sequence-specific and dose dependent. In one case the ODN was more efficient than a previously described siRNA directed against the same target site in the mRNA of UL5, a component of the helicase/primase complex. HSV-1 virions and ODNs can be applied simultaneously without transfection reagent, but at a 50-fold higher concentration to Vero cells with similar efficiencies. The results underline the potential of partially double-stranded hairpin loop-structured ODNs as antiviral agents.

## Findings

**Figure 1:**
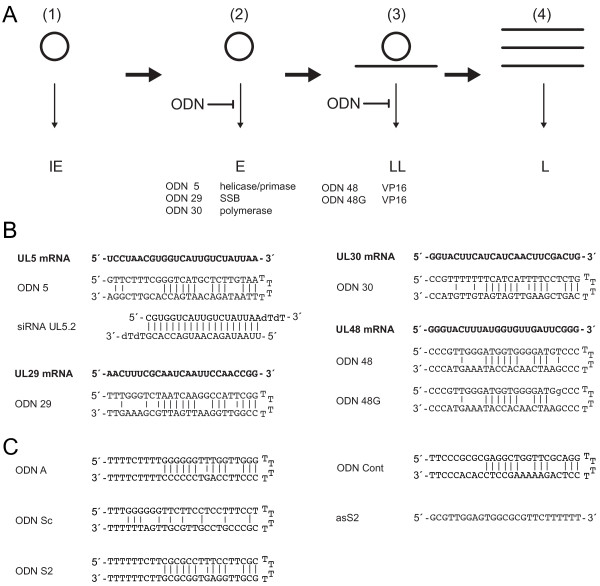
**Model for ODN-mediated inhibition of HSV-1 and ODNs used**. A. The HSV expression cascade during replication consists of the expression of (1) immediate early genes (IE) and (2) early (E) genes from the circularized HSV genome, of (3) leaky late genes (LL) from the parental genome and progeny genomes and of (4) late genes (L) from progeny genomes. ODNs target mRNAs of early and leaky late gene products, a component of the helicase/primase complex, the single strand binding protein (SBB), the polymerase as well as the tegument protein VP16, which is a component of the virion and facilitates IE gene expression in the next round of infection. B. Target sites (bold) and sequences of the oligonucleotides used. C. ODN A, S2, Sc, Cont and asS2 served as negative controls.

The Herpes simplex virus (HSV), a member of the family Herpesviridae, is a large DNA virus with a high prevalence in the human population. HSV genes are expressed in a highly regulated cascade during productive infection [1]. They are grouped in immediate early (IE), early (E), leaky-late (LL) and late genes (L) according to the time point of expression after infection (figure 1A). In this study, we are targeting four essential HSV-1 genes with a new class of oligodeoxynucleotides, partially double-stranded hairpin loop-structured 54 mer ODNs. The ODNs consist of a 25 mer antisense strand fully complementary to the target mRNA site, a linker of four thymidines, and a 25 mer second strand partially complementary to the antisense strand. ODNs with the same structure proved their capability to mediate hydrolysis of HIV-1 RNA by HIV-1 reverse transcriptase (RT)-associated RNase H in HIV virions and patient-derived plasma in a sequence specific manner as shown before *invitro* and in animal studies [2-9].

The sequences of the antisense strands of the ODNs used here were chosen on the basis of known siRNA sequences, recently shown to significantly inhibit HSV replication [10,11]. The structure, sequences, and target sites of the ODNs are shown in figure 1B. ODN 5, 29, and 30 target the mRNA of the E genes *UL 5*, *UL 29*, and *UL 30 *essential for DNA synthesis [12]. *UL 5 *encodes a component of the helicase/primase complex, *UL 29 *a single strand binding protein and *UL 30 *the DNA polymerase. ODN 48 and 48 G target the mRNA of the LL gene *UL 48*, which encodes the virion associated VP16 needed in progeny virions for the transcriptional activation of IE genes in the next round of infection [13]. As controls, we used different oligodeoxynucleotides without any sequence similarity to the HSV-1 genome (figure 1C) or phosphate buffered saline (PBS). The ODNs used were protected against nucleases by thioate modifications of the three terminal nucleotides at each end and in the T4 linker as described [6,14].

We have chosen African green monkey kidney (Vero) cells and human embryonic lung fibroblast (MRC-5) to demonstrate the effect of the ODNs on HSV type 1 (HSV-1) replication *in vitro*. Both cell lines are permissive to HSV-1 strain McIntyre infection. Vero cells are defective in interferon production and HSV infections of monolayers produce a clear plaque phenotype. The MRC-5 cells were chosen to confirm the results in a primary human cell line.

**Figure 2:**
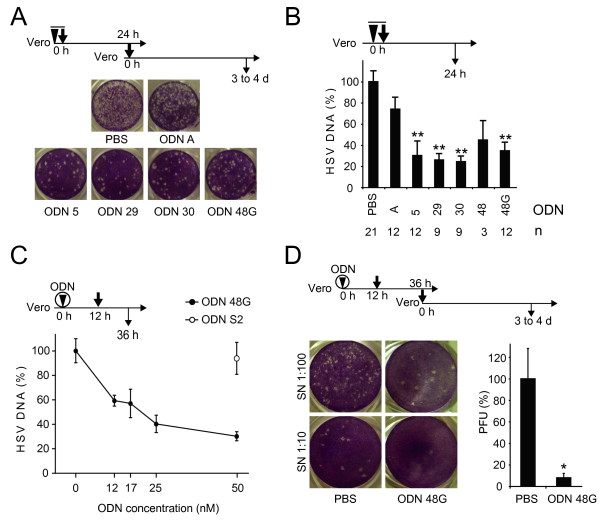
**Inhibition of HSV-1 by ODNs**. A and B. Co-application of ODNs and HSV-1. HSV-1 virions were mixed with 2.5 μM of the indicated ODNs, a concentration needed in the absence of transfection reagent. The mixture was added to Vero cells (arrow head and bold arrow with overline). The titer of HSV-1 was determined after 24 h. A. Plaque assay. The supernatants of infected cultures were assayed for plaque formation on Vero cells. Representative 1:100 dilutions of triplicates are shown. B. Quantitative PCR. HSV-1 DNA was purified from supernatants and quantified by glycoprotein G-specific PCR. Relative HSV-1 DNA levels are shown +SE. The total number of infections (n) for each ODN is indicated below the graph. The statistical significance is indicated by asterisks, **P ≤ 0.01 against PBS. C. Vero cells were treated with various concentrations of ODN 48 G and S2 as indicated in the presence of transfection reagent (indicated by circle) at the indicated concentrations and were incubated over night (o/n) at 37°C. The cells were infected (bold vertical arrow) at a moi = 0.001 and after 24 h the HSV-1 DNA levels in the supernatants were measured by quantitative PCR (vertical arrow). The mean value ± SE of 2 different experiments is shown. D. Plaque Assay. Vero cells were transfected with 50 nM ODN 48 G or phosphate-buffered saline (PBS) and infected as described in A. Dilutions of the supernatant were assayed for plaque formation on Vero cells. Representative pictures of triplicates are shown. *P ≤ 0.05 against PBS.

First, we screened for an antiviral effect in confluent Vero cells. The media were replaced by Dulbecco's modified Eagle Medium (DMEM) with 2% fetal calf serum (FCS) containing a mixture of HSV-1 virions and ODNs for co-application. The virus was introduced at a multiplicity of infection (moi) of 0.001 and the final concentration of ODNs was 2.5 μM. After 24 h the cell culture supernatants were harvested and HSV DNA levels were determined by quantitative PCR (qPCR) using primers and a probe targeting the HSV-1 glycoprotein G [15]. The ODNs against HSV-1 reduced the viral titer by 60 to 80% in comparison to infected cells incubated with PBS (data not shown). To confirm this result the supernatants from this experiment were serially diluted and used to infect confluent Vero cells for plaque assays (figure 2A). After 1 h of incubation with the virus the cells were overlaid with DMEM containing 2% FCS and 0.4% noble agar and grown for 3 to 4 days at 37°C. After removal of the medium the cells were washed with PBS and stained with 1% crystal violet in 20% ethanol. The ODNs against HSV-1 exhibited a significant reduction of the viral titer, whereas the observed effect with the control ODN A was comparable to the infected cells treated with PBS as negative control. We performed several experiments with co-application under the same conditions and measured the viral titer in cell culture supernatants by qPCR 24 h after co-application. The result is summarized in figure 2B. A two-tailed Student's t-test with unequal variance was used to perform statistical analysis. The reduction of viral titers was statistically significant for ODNs 5, 29, 30 and 48 G (p < 0.01 against PBS), but not for ODN 48 and the control ODN A. The HSV-1 DNA levels could be reduced by 75%, although HSV replication was slightly reduced by the control ODN A in comparison to cells that were infected without ODNs.

**Figure 3:**
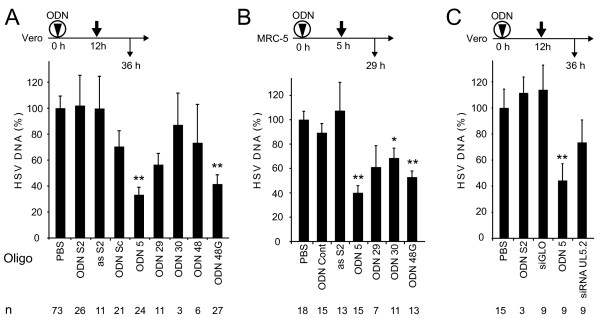
**Analysis of ODN-mediated reduction of HSV-1 titer in Vero cells and primary lung fibroblasts**. A, B and C. Transfection of ODNs. Vero cells (A, C) and primary lung fibroblasts (B, MRC-5 cells) were treated with 50 nM of the indicated ODNs or PBS for 5 to 16 h in the presence of transfection reagent. The medium was changed to DMEM and cells were incubated for 1 h. The cells were infected and after 24 h the HSV-1 DNA levels in the supernatants were determined by quantitative PCR. The mean value of all experiments +SE is shown. The total number of infections performed (n) is indicated below the graph. **P ≤ 0.01, *P ≤ 0.05 against PBS. C. Comparison of ODN 5 and siRNA UL5.2 targeting the same region of UL5 mRNA, performed as described in A. As negative control the commercially available siRNA siGLO RISC-Free (Dharmacon) was used.

In order to examine the effect in more detail, we performed experiments in Vero and MRC-5 cells using transfection reagents, in contrast to the above-mentioned experiment. To establish the best conditions for transfection, we determined transfection rates for a fluorescein-labelled ODN A with different transfection reagents in Vero cells. The best transfection rates were achieved after an overnight incubation with Lullaby or Dreamfect Gold transfection reagents, which were consecutively used in further experiments (data not shown). Then, we transfected Vero cells with different concentrations of ODN 48 G and incubated the cells overnight, before they were subsequently infected with a moi of 0.001. Cells treated with the unspecific ODN S2 at a final concentration of 50 nM or PBS served as controls. 24 h after the infection the HSV DNA levels in cell culture supernatants were determined by qPCR. The reduction of viral replication was dose dependent with a plateau at a concentration of 50 nM (figure 2C). The control ODN S2 did not show a reduction of viral titer at this concentration. The supernatants of the cells treated with ODN 48 G at a concentration of 50 nM and the cells treated with PBS were used for plaque assays. A reduction of plaque forming units by 90% in comparison to cells treated with PBS was observed (figure 2D). Transfection reagents thus allow a reduction of the ODN concentrations. Further transfection studies were carried out with ODNs at 50 nM concentrations. Figure 3A displays a summary of all experiments with transfection of ODNs into Vero cells and subsequent infection after 12 h. The control oligonucleotides, single-stranded asS2, and scrambled ODN Sc were tested additionally to ODN S2 to investigate the sequence specifity of the observed effects. Only ODN Sc showed a slight reduction of the HSV-1 DNA levels, but this effect was not significant. ODN 5 reduced the HSV-1 DNA levels significantly by 70% (p < 0.01) and ODN 48 G by 60% (p < 0.01). The ODNs 29, 30 and 48 did not show a significant reduction of the titer. A cytotoxic effect of the ODNs was not observed as revealed by proliferation assays (data not shown).

To examine whether the effect is reproducible in other cell lines such as a primary human cell-line, we transfected MRC-5 cells with the ODNs at a final concentration of 50 nM. The cells were infected 5 h post transfection at a moi of 0.001 for 1 h and the viral titer was assayed 24 h after infection by qRT-PCR. HSV-1 replication was only impaired in cells treated with the HSV-specific ODNs (figure 3B). The controls ODN Cont and asS2 were negative. The effect was statistically significant for ODN 5, 30 and 48 G. The ODNs did not exhibit a cytotoxic effect in MRC-5 cells (data not shown). A direct comparison between ODN5 and an siRNA is shown for Vero cells in figure 3C.

Table 1 summarizes all experiments listed under the different conditions. Overall, ODN 5, targeting a component of the helicase/primase complex, has the greatest potential to inhibit HSV-1 replication *in vitro*, which is consistent with reports about the helicase/primase complex being a target for inhibiting the HSV-1 replication by siRNA [10] (see figure 3C) and antiviral compounds [16,17].

**Table 1:** Reduction of HSV-1 DNA levels upon treatment with ODNs in Vero and MRC-5 cells.

Treatment	Average fold reduction of the mean (median)		
	
	Co-application	Transfection with subsequent infection	
		
	Vero	Vero	MRC-5
PBS	1.0 (1.0)	1.0 (1.0)	1.0 (1.0)
Control	1.4 (1.4)	1.0 (1.0)	1.1 (1.1)
ODN 5	3.3 (6.5)	3.0 (5.0)	2.5 (2.2)
ODN 29	3.9 (4.1)	1.8 (1.3)	1.6 (2.4)
ODN 30	4.1 (4.1)	0.7 (1.3)	1.5 (1.6)
ODN 48	2.2 (3.1)	1.4 (1.7)	n. d.
ODN48G	2.9 (3.6)	2.4 (2.4)	1.9 (2.0)

The data of all *in vitro *experiments are summarized. The average fold reductions of the mean and the median are shown (n. d., not determined).

Antisense ODNs and small interfering RNAs are established antiviral agents that have been shown to reduce HSV replication [10,11,18]. Here we are demonstrating that a new class of oligodeoxynucleotides – partially double-stranded hairpin loop-structured ODNs with one strand completely complementary to the target mRNA – can reduce HSV-1 replication *in vitro*. The result is consistent with previous studies reporting that hairpin loop-structured ODNs have an antiviral effect on HIV-1 and Influenza virus [2-9,19]. The modes of action of the ODNs used in this study may be due to steric hindrance of ribosomes and hybridization to the corresponding mRNA thereby creating a substrate for cellular RNases H, comparable to the modes of action of single-stranded ODNs [20]. In a direct comparison with siRNAs and ODNs in Vero cells both oligonucleotides were differentially effective, e.g. the most effective ODN 5 in this study was 2.5-fold more effective than the corresponding siRNA-UL5.2 [10] (figure 3C), whereas ODN 29 was 2-fold less effective than its analog (data not shown). This suggests that different target sites might have preferences for particular types of oligonucleotides. Furthermore, the second strand linked to the antisense strand can modulate the effect in a positive or negative manner by affecting stability, accessibility, localization or uptake of the ODN. Overall, this study underlines the potential of partially double-stranded hairpin loop-structured ODNs as antiviral agents.

## Competing interests

The authors declare that they have no competing interests.

## Authors' contributions

AF performed the experiments. JH participated in the design of the study, designed the ODNs and helped performing the experiments. KM initiated the study. AF, JH and KM analyzed the data and wrote the manuscript. All authors read and approved the final manuscript.
